# Empagliflozin’s role in cardiometabolic disease treatment: an Egyptian expert’s view

**DOI:** 10.1186/s13098-026-02312-y

**Published:** 2026-10-30

**Authors:** Samir H. Assaad-Khalil, Fawzy Elmessallamy, Khaled Elsayed Elhadidy, Mohamed Hesham El Hefnawy, Mohamed Reda Halawa, Mohamed Zeitoun, Shady Hussein, Ibrahim Elebrashy

**Affiliations:** 1 Unit of Diabetes, Lipidology & Metabolism, Department of Internal Medicine, Faculty of Medicine, Alexandria University, Alexandria, Egypt; 2 Diabetes and Endocrine Unit, Department of Internal Medicine, Faculty of Medicine, Zagazig University, Zagazig, Egypt; 3 Diabetes and Metabolism Unit, Department of Internal Medicine, Faculty of Medicine, Beni-Suef University, Beni-Suef, Egypt; 4 National Institute of Diabetes and Endocrinology, Cairo, Egypt; 5 Department of Endocrinology, Ain Shams University, Cairo, Egypt; 6 Department of Cardiology, Mansoura University, Mansoura, Egypt; 7 Department of Internal Medicine and Diabetes, Cairo University, Cairo, Egypt; 8 Department of Internal Medicine, Unit of Diabetes, Lipidology, and Metabolism, Faculty of Medicine, Alexandria University, Alexandria Governorate, Alexandria, Egypt

**Keywords:** Diabetes mellitus, Empagliflozin, Hypertension, Metabolic dysfunction-associated steatotic liver disease, Ramadan, SGLT-2 inhibitor

## Abstract

**Background:**

Given the expanding therapeutic indications of empagliflozin (EMPA) beyond glycemic control and the growing burden of cardiometabolic diseases, this review evaluated its efficacy, safety, and cost-effectiveness in the Egyptian population within the global context.

**Methods:**

A comprehensive literature review of PubMed and Google Scholar identified clinical trials, systematic reviews, meta-analyses, and clinical guidelines published in English up to July 2025. We included studies reporting EMPA’s pharmacological profile, mechanisms of action, and clinical outcomes on diabetes mellitus, cardiometabolic diseases, renal and hepatic functions, safety during Ramadan fasting, and cost-effectiveness.

**Results:**

EMPA, as monotherapy or combination therapy, improves glycemic control in type 2 diabetes through Sodium–glucose co-transporter-2 (SGLT-2) inhibition. For type 1 diabetes mellitus, despite improving glycemic control as an adjunct therapy, it is not recommended due to the increased risk of diabetic ketoacidosis. EMPA reduces major cardiovascular events, heart failure hospitalization, and chronic kidney disease progression, with modest weight loss and blood pressure reduction, in patients with or without diabetes. Emerging evidence suggests benefits in metabolic dysfunction-associated steatotic liver disease (MASLD), although it is not approved for this indication. EMPA demonstrates a favorable safety profile and low hypoglycemia risk during Ramadan fasting. It enhances patients’ quality of life and is considered cost-effective.

**Conclusions:**

EMPA extends beyond glucose-lowering by reducing cardiorenal morbidity and mortality in patients with or without diabetes. It shows a favorable safety profile, even during Ramadan fasting, supporting its suitability for the large Muslim population. Further research should evaluate its role in MASLD and assess cost-effectiveness in Egypt.

**Graphical abstract:**

**Figure:**
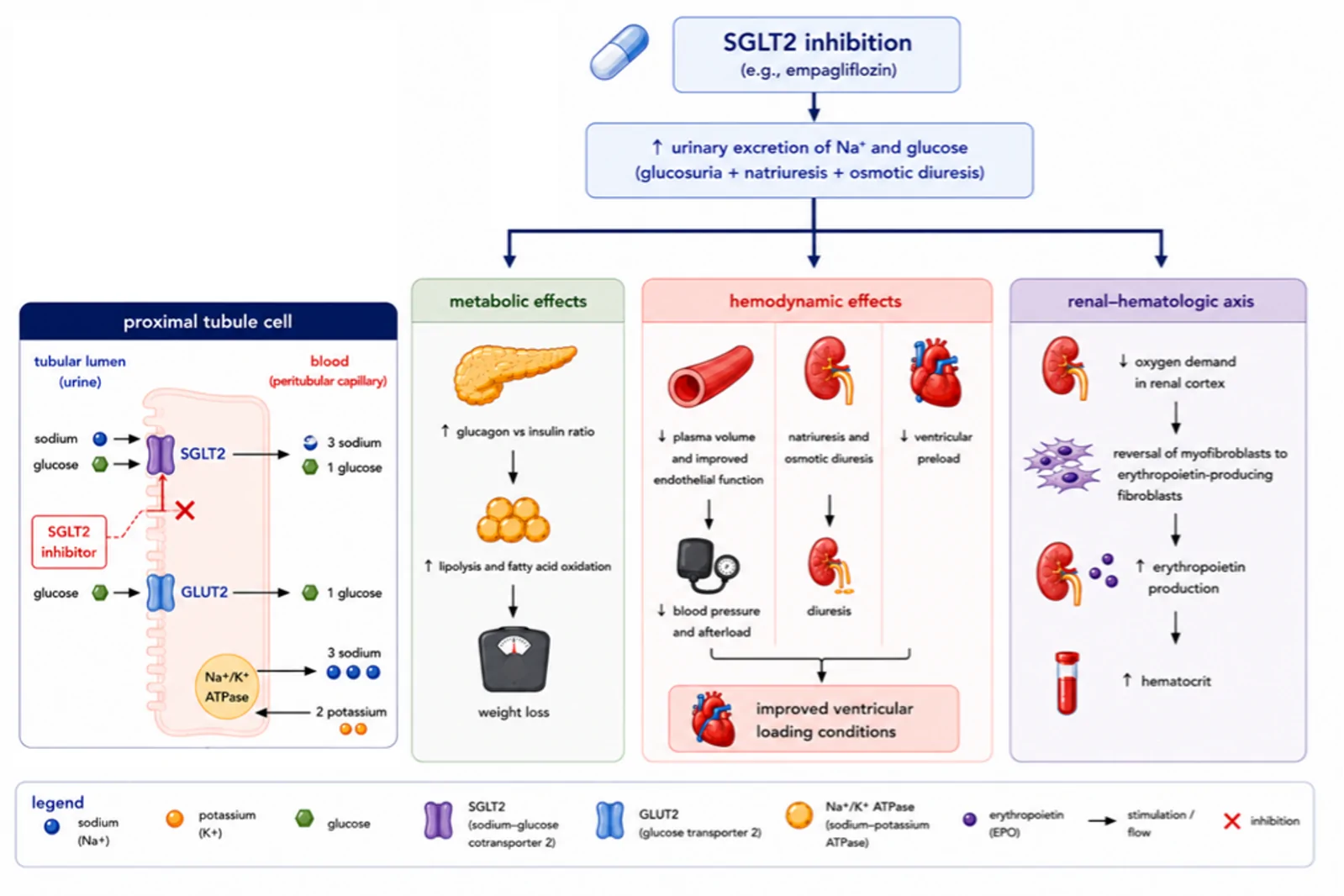

## Introduction

Diabetes Mellitus (DM), the epidemic of this era, is a major global health burden. The International Diabetes Federation estimates that 589 million people aged 20–79 currently live with DM, a number projected to reach 853 million by 2050 [1]. The World Health Organization reported that, in 2021 alone, DM and its complications led to over two million deaths, ranking DM as the 8th leading cause of death globally [2, 3]. In 2024, Egypt had an estimated adult population of 66.6 million, among whom 13.2 million individuals were living with diabetes, corresponding to a prevalence rate of 22.4% [4]. A pooled global analysis flagged Egypt as one of the countries in the Middle East and North Africa (MENA) region with the largest increases in age-standardized diabetes prevalence between 1990 and 2022 [5].

Both type 1 type 1 and type 2 diabetes mellitus (T1DM and T2DM) are associated with a greater risk of cardiovascular diseases (CVD), including stroke, heart failure (HF) [6, 7], myocardial infarction (MI), and other ischemic heart disease [8, 9]. DM-associated CVD doubles the mortality risk and shortens life expectancy by ~ 12 years [10]. Recently, the prevalence and clinical management of atherosclerotic cardiovascular disease (ASCVD) in patients with T2DM across countries in the Middle East and Africa study (PACT-MEA) reported that, in Egypt, the prevalence of established ASCVD was 19.6%, including 15.1% with coronary artery disease, 3.1% with CVD, and 2.9% with peripheral artery disease. Among individuals without established ASCVD, an additional 85.5% were estimated to be at high ASCVD risk according to the European Society of Cardiology (ESC) classification [11].

The burden of metabolic dysfunction–associated steatotic liver disease (MASLD) and its progressive form, metabolic dysfunction-associated steatohepatitis (MASH), is rising globally in parallel with obesity and diabetes [12]. In the MENA region, MASLD prevalence is estimated at 36.5%, with Egypt projected to harbor 25.7 million cases and modeling studies suggesting > 40–45% prevalence in the general population [13]. Among young Egyptian adults, prevalence is 31.6% [14]. Progression from MASLD to MASH drives fibrosis, cirrhosis, and hepatocellular carcinoma, with MASLD already implicated in 12.8% of cirrhosis-related deaths in Egypt [15].

Empagliflozin (EMPA), a Sodium-Glucose co-transporter-2 (SGLT-2) inhibitor (SGLT-2i), decreases glucose renal reabsorption, leading to a simultaneous increase in glucose urinary excretion. Thus, the medication’s ability to reduce blood sugar is entirely insulin-independent. In clinical practice, EMPA is sometimes used in combination with glucagon-like peptide-1 receptor agonists in patients with T2DM, particularly those with obesity or MASLD; however, these indications remain off-label use. Recent trials and meta-analyses reported that EMPA can reduce liver enzyme levels (ALT, AST, GGT) and hepatic steatosis in T2DM cohorts with nonalcoholic fatty liver disease (NAFLD) [16–18]. In patients with obesity, treatment with EMPA resulted in a significant reduction in body weight to 91.9 ± 18.0 kg (*P* = 0.001) in Egypt [19].

**Fig. 1:**
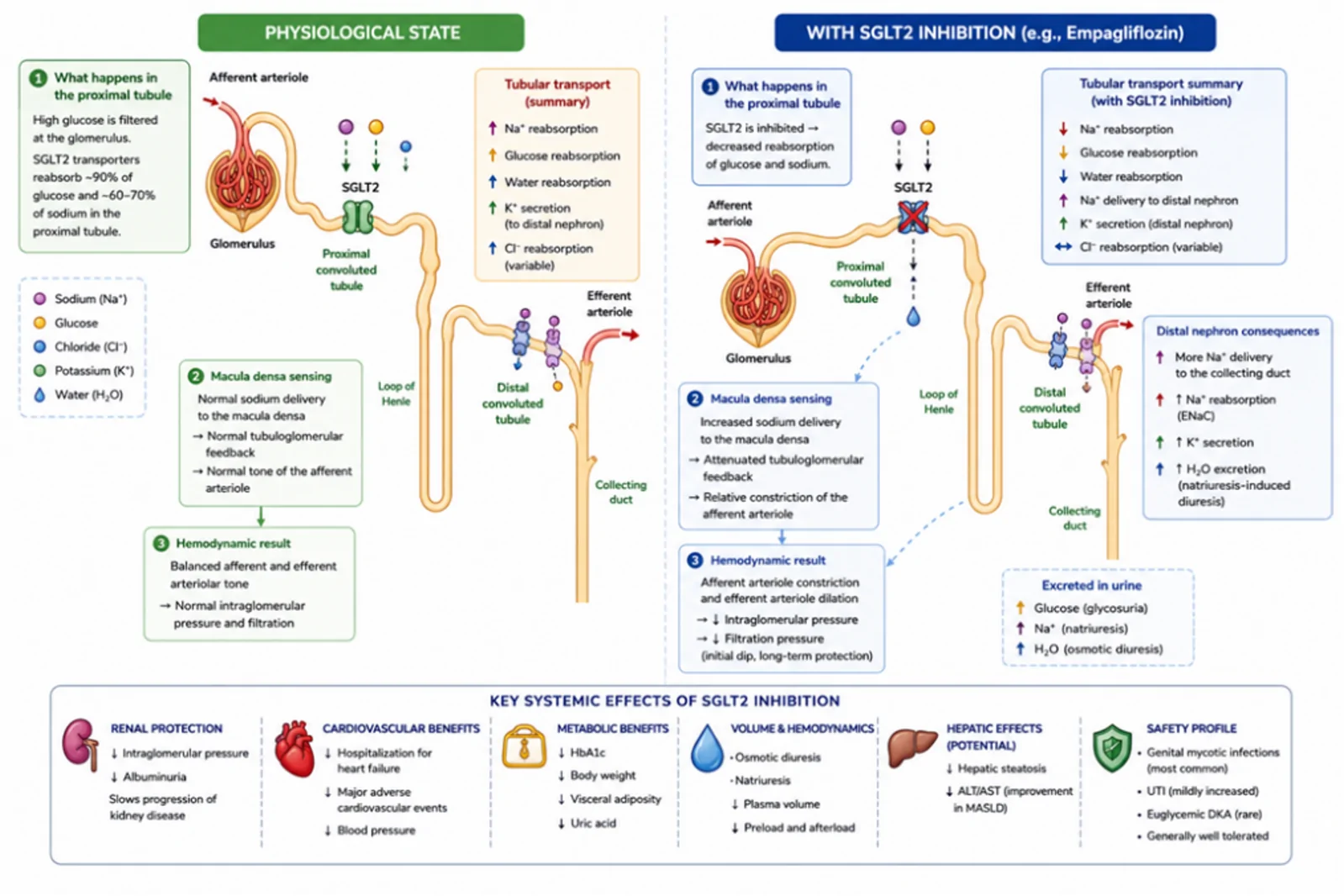
Beneficial effect of empagliflozin on the body organs. SGLT2, sodium–glucose cotransporter 2; GLUT, glucose transporter; Na⁺, sodium ion; K⁺, potassium ion; ATPase, adenosine triphosphatase; ATP, adenosine triphosphate

EMPA is formulated as oral tablets in 10 mg and 25 mg strengths, administered once daily. The dosage may be titrated to 25 mg if clinically indicated [20]. EMPA reduces sodium and volume load *via* diuretic and natriuretic effects, promoting weight loss and lowering blood pressure (BP) without increasing heart rate. It reduces hospitalization for HF in patients with left ventricular (LV) dysfunction, acute MI, or chronic cardiovascular disease, and slows progression of kidney disease [21–23] while reducing liver lipid content **(**Fig. 1**)** [24]. In Egypt, EMPA offers an available therapeutic option for patients with HF and reduced ejection fraction, regardless of diabetes status. EMPA demonstrated cardiovascular and renal benefits in clinical trials, along with a favorable safety profile, positioning it as a practical addition to standard HF management in eligible Egyptian patients [25]. Accordingly, the American Diabetes Association, European Association for the Study of Diabetes, ESC, and Kidney Disease: Improving Global Outcomes (KDIGO have recommended the use of EMPA in patients with cardiometabolic diseases to improve outcomes and decrease mortality [24, 26]. Despite established benefits, evidence is conflicting for its use in T1DM, NAFLD, and during prolonged fasting, such as Ramadan, warranting safety evaluation. Furthermore, although international studies suggest EMPA is cost-effective due to long-term reductions in cardiovascular and renal hospitalizations, pharmacoeconomic data required for local healthcare policy in Egypt remains scarce [27–29]. 

This comprehensive narrative review aimed to synthesize global and localized Egyptian evidence on EMPA’s mechanism of action, pharmacology, clinical efficacy across various cardiometabolic diseases (with or without diabetes), safety profile, cost-effectiveness, quality of life (QoL), and use during Ramadan fasting.

## Methods

This narrative review was based on a comprehensive, structured literature search of the PubMed and Google Scholar databases for relevant articles published up to July 2025. The search utilized combinations of keywords and Boolean operators, including terms such as “empagliflozin”, “SGLT-2 inhibitors”, “pharmacology”, “mechanism of action”, “type 2 diabetes mellitus”, “type 1 diabetes mellitus”, “cardiovascular outcomes”, “heart failure”, “chronic kidney disease”, ”nephroprotection”, “weight loss”, “blood pressure”, “Metabolic Dysfunction-Associated Steatotic Liver Disease”, “hepatic steatosis”, “Ramadan”, “fasting” “adverse effects”, “safety”, “cost-effectiveness”.

Studies were included if they involved human subjects and were published in English. Articles were excluded if they were in vitro or animal studies, single case reports, expert opinions without data synthesis, or published in languages other than English. This review prioritized landmark randomized controlled trials, large-scale meta-analyses, systematic reviews, major clinical guidelines, and health economics studies. Evidence was evaluated qualitatively according to study design, sample size, population characteristics, clinical relevance, methodological rigor, and consistency with other available evidence. When conflicting evidence was encountered, discrepancies were managed by comparing designs, patient characteristics, and specific inclusion criteria.

## Results

### Mechanism of action

Chronic hyperglycemia can cause β-cell death without neogenesis. Uncontrolled diabetes is believed to cause glucosuria, but inhibiting renal SGLT-2 can cause glucose excretion [30]. Growing evidence suggests tubular factors contribute to hyperfiltration in patients with diabetes, resulting in increased SGLT-2 mRNA expression [31]. EMPA is one of the globally approved SGLT-2i, alongside dapagliflozin and canagliflozin [32].

EMPA has demonstrated a renoprotective effect through an increase in distal sodium delivery, leading to adenosine release and afferent vasoconstriction. This has led to continuous urinary glucose loss of 40–80 g per day.

The clinical benefits of EMPA extend beyond glycemic control and kidney protection, encompassing cardiovascular and metabolic benefits. One meta-analysis has concluded that diabetic patients with HF are more prone to all-cause death, CV death, and hospitalization [33]. The reduction of CVD risk is attributed to the amelioration of transforming Growth Factor Beta (TGF-β), Adenosine Triphosphate (ATP), and Reactive Oxygen Species (ROS) [34].

**Fig. 2:**
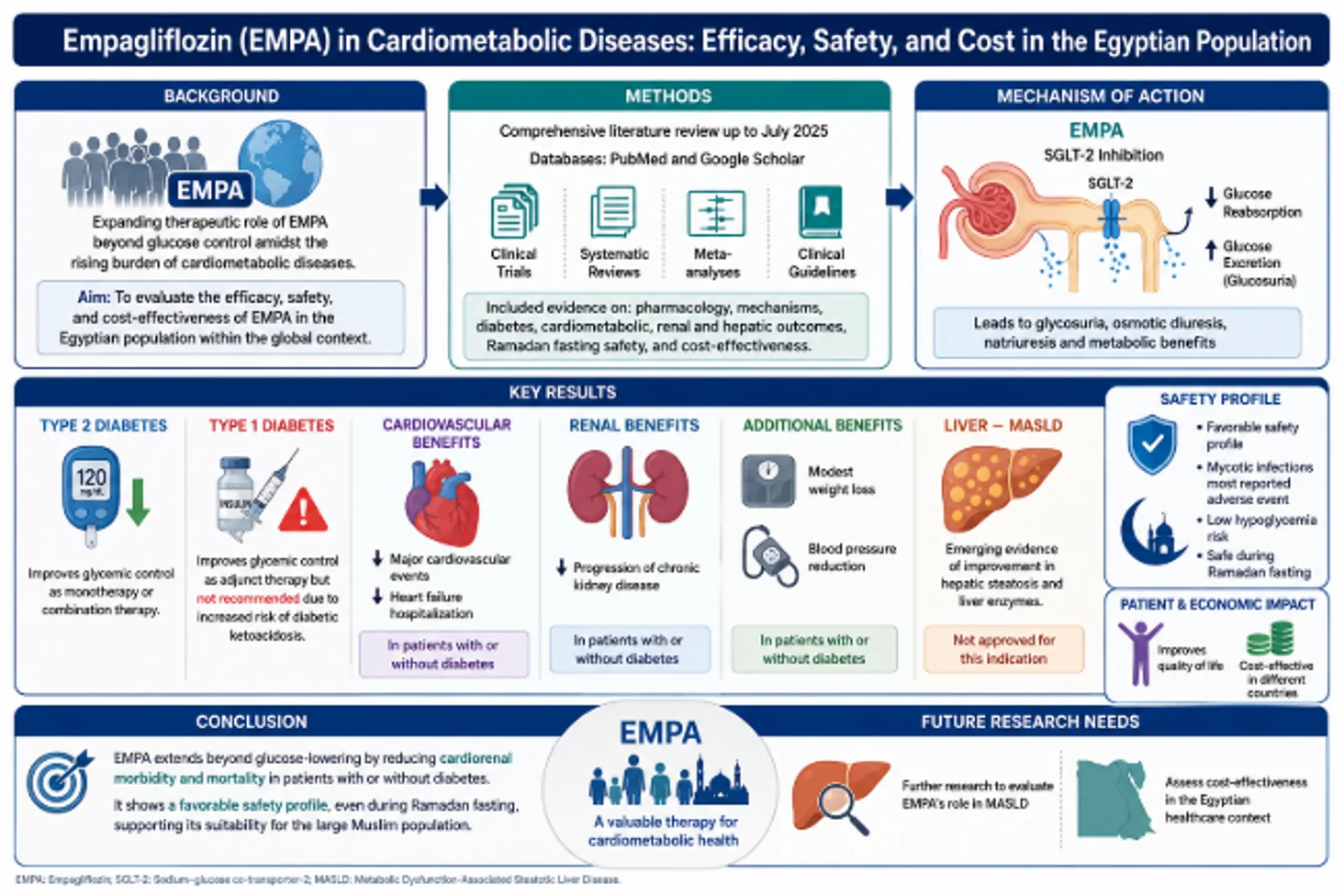
Mechanism of action for empagliflozin. ALT, alanine aminotransferase; AST, aspartate aminotransferase; DKA, diabetic ketoacidosis; ENaC, epithelial sodium channel; HbA1c, glycated hemoglobin; HF, heart failure; H₂O, water; K⁺, potassium; MASLD, metabolic dysfunction–associated steatotic liver disease; Na⁺, sodium; SGLT2, sodium–glucose cotransporter 2; UTI, urinary tract infection

One proposed mechanism by which SGLT-2i attenuates glomerular hyperfiltration is through increased sodium delivery to the macula densa, which restores tubuloglomerular feedback and reduces single-nephron glomerular filtration rate (SNGFR). During high blood glucose episodes, a 70% reduction in proximal sodium reabsorption occurred along with a critical drop in SNGFR [35]. Furthermore, in the T1DM Akita mouse model, EMPA showed a similar impact on accompanying hyperfiltration with preservation of glomerular size, kidney mass, and kidney mRNA expression of inflammatory biomarkers such as Tissue Inhibitor of Metalloproteinase-2 (TIMP-2), monocyte chemotactic protein-1 (MCP-1), and IL-6 [36], suggesting both direct and indirect beneficial effects of EMPA in preventing deterioration of organs affected by DM **(**Fig. 2**)**.

### EMPA in patients with T2DM

EMPA, administered once daily as an oral agent, exerts its effects through an insulin-independent mechanism, which minimizes the inherent risk of hypoglycemia. This property allows its use both as monotherapy and in combination with other antidiabetic therapies that have complementary mechanisms of action [37, 38]. Evidence from multinational, double-blind, placebo- and active-controlled phase III trials, enrolling 333–1549 individuals with T2DM and lasting 12 to 104 weeks, has consistently shown that EMPA at doses of 10 or 25 mg/day significantly lowers HbA1c compared with placebo, whether used alone or as add-on therapy [39]. Parallel reductions in fasting plasma glucose were also observed in these studies.

Across several trials and their long-term extensions, empagliflozin not only improved glycemic parameters but also contributed to reductions in body weight and systolic BP. When combined with metformin, it demonstrated noninferiority to glimepiride at 52 and 104 weeks and superiority by week 104 in terms of HbA1c reduction [37]. In Egypt, EMPA has shown superior efficacy in improving glycemic control, reducing body weight, and lowering BP in patients with T2DM uncontrolled on metformin [19].

### EMPA and cardiovascular events

T1DM and T2DM are both associated with cardiovascular conditions, including coronary heart disease, stroke, peripheral arterial disease, cardiomyopathy, and congestive HF. The American Heart Association identifies diabetes as a predictive factor for CVD [39] in addition to the increased prevalence of smoking, high BP, and cholesterol disorders [40]. These risk factors are major predictors of CVD among Egyptian patients [41–43]. Angiotensin II and hyperglycemia increase oxidative stress, leading to adverse cardiac remodeling and LV ejection fraction dysfunction. This pathological condition was found to be effectively blocked by SGLT-2i [44]. CVD risk is higher when DM and hypertension coexist than with either condition alone [45]. The Canadian Cardiovascular Society and the Canadian Heart Failure Society recommended the use of SGLT-2i in preventing or managing patients with HF with or without DM [46].

In the EMPA-REG trial, EMPA reduced HF hospitalizations and mortality in T2DM [47] and lowered ischemic stroke incidence and all-cause mortality compared with dipeptidyl peptidase-4 inhibitors [48]. It improved LV end-diastolic and end-systolic volumes without changing LV or Right ventricular (RV) functional parameters [49]. The EMPA-HEART study found reduced LV mass index in T2DM with coronary artery disease [50].

In the EMPACT-MI trial, the primary endpoint was a composite of hospitalization for HF or death from any cause. In a pre-specified analysis of this trial, EMPA reduced first HF hospitalizations by 23% and total hospitalizations by 33%, but not all-cause mortality, in acute MI patients with LV ejection fraction < 45% and/or congestion, irrespective of T2DM status [22, 51]. In patients with ASCVD with T2DM, EMPA reduced MI risk [52] but did not affect high-sensitivity C-reactive protein (hs-CRP) in patients undergoing percutaneous coronary intervention [53]. In Egyptian patients with T2DM and coronary artery disease, EMPA has demonstrated significant cardiovascular benefits, improving atrial function, ejection fraction, and cardiac structural parameters. These findings emphasize its role not only in glycemic management but also in early cardioprotection, supporting its integration into diabetes care to reduce cardiovascular burden in Egypt [54].

However, other studies found no change in left atrial volume after six months of EMPA in T2DM [55], and no improvement in LV systolic dysfunction post-MI with standard care [56]. Similarly, the EMPACT-MI trial showed no reduction in hospitalization or death in high-risk post-AMI patients [57].

Conversely, another trial reported a dose-dependent improvement in QT interval and ventricular repolarization [58], and Fujiki et al. observed fewer ventricular arrhythmias during EMPA treatment compared with baseline [59]. A recent meta-analysis estimated that EMPA and Dapagliflozin reduced the risk of sudden cardiac death by 18% in patients with diverse cardiovascular risk profiles [60]. Moreover, EMPA substantially elevated blood ketones and hematocrit with a decline in blood-brain natriuretic peptide and body weight [61]. This heterogeneity in results could be due to slower effects developed on long-term treatment with EMPA, where the acute MI patients won’t benefit from them.

Overall, the cardiovascular outcomes of EMPA are supported by clinical evidence; however, the benefits should be interpreted in the context of the patient population and specific clinical outcomes assessed.

### EMPA and BP

Hypertension is considered a major predictive factor for CVD, which is commonly linked to DM [62]. In T2DM with hypertension, EMPA lowered systolic BP by 8 mmHg, and when combined with losartan, by 15 mmHg. EMPA also reduced sympathetic activity, arterial stiffness, and extracellular fluid, while increasing serum albumin [63]. In T2DM patients with hypertension, EMPA plus adjuvant treatment markedly enhanced QoL as well as BP and glucose indicators [64]. EMPA reduced brachial, central ambulatory BP, and arterial stiffness across patients with or without T2DM and CKD [65]. In Egyptian patients with T2DM, baseline mean systolic and diastolic BP in the EMPA group were 136.7 ± 17.8 mmHg and 85.6 ± 10.8 mmHg, respectively. After 12 weeks of therapy, empagliflozin decreased systolic BP to 132.1 ± 12.9 mmHg (*P* = 0.016) and diastolic BP to 82.9 ± 8.3 mmHg (*P* = 0.006). Although between-group differences did not reach statistical significance (*P* = 0.203 and 0.041, respectively), the results indicated that EMPA contributes to improved BP control and favorable hemodynamic effects in Egyptian diabetic patients [19].

In older diabetics with uncontrolled nocturnal hypertension, EMPA reduced nighttime ambulatory systolic BP [66, 67]. Diabetic patients with hypertension showed improved random blood sugar, brachial and central BP, estimated glomerular filtration rate (eGFR), and albuminuria without significant changes in pulse rate or lipid profiles [68]. Similarly, EMPA declined BP, body weight, and HbA1c in black patients with T2DM after 12 weeks of therapy, despite full EMPA effect could not be observed unless 6 months of therapy had been achieved [69].

The available evidence suggests that EMPA is associated with modest reductions in blood pressure, the magnitude of which varies with patient characteristics and treatment context.

### EMPA in kidney dysfunction

The prevalence of diabetic nephropathy is rising in Egypt, with around 36.5% of diabetic patients at risk of developing chronic kidney disease (CKD) [61]. SGLT-2i are assumed to play a vital role in diabetic nephropathy through the theory of changes in renal hemodynamic function. The theory demonstrates a protective role of SGLT-2i by decreasing the reuptake of extremely filtered sodium and glucose, causing an increase in sodium transport to the macula densa [70].

A multinational consensus (Egypt, Mexico, Singapore, South Korea) identified early SGLT-2i use as crucial for slowing CKD progression in non-diabetic patients and delaying CKD onset and progression in T2DM with HF [71]. In the EMPA-KIDNEY trial, a 10-mg daily dose of EMPA significantly decreased the risk of CKD progression in non-diabetic patients to 23.5% compared to 27.1% in the placebo group [72]. A subsequent meta-analysis pooling data from 23,340 participants across four large trials, EMPA-REG OUTCOME, EMPEROR-Reduced, EMPEROR-Preserved, and EMPA-KIDNEY, showed that EMPA reduced AKI adverse events by 27%, CKD progression by 30%, and kidney failure by 34% compared to placebo. These benefits were independent of diabetes or HF status, baseline kidney function, or albuminuria level [73]. Other studies have emphasized the role of EMPA in decreasing cardiovascular events and kidney function deterioration in patients with CKD [74–77]. EMPA also reduced BP, total vascular resistance, and GFR in both diabetic and non-diabetic patients [78]. EMPA also lowers uric acid levels and kidney stone risk [79]. For instance, the EMPEROR-reduced trial shed light on the key role of EMPA in reducing serum uric acid and hyperuricemia in those patients [79]. EMPA also reduced the level of uric acid and gout episodes in patients with T2DM [80]. This could be due to the efficacy of EMPA in decreasing urine relative supersaturation ratios for calcium oxalate and calcium phosphate by 36% relative to placebo [81].

EMPA use was associated with lower kidney injury biomarkers (neutrophil gelatinase-associated lipocalin, kidney injury molecule-1) and improved function, reflected in reduced urine albumin-to-creatinine ratio (UACR) and increased eGFR [82]. EMPA also lowered cystatin C levels and reduced fluid overload on bioimpedance, regardless of age [83]. Notably, a reduction in fluid overload was found to be effective in treating conditions such as inappropriate antidiuresis, where hyponatremia and fluid retention occur, leading to improved neurocognitive function in those patients [84]. Moreover, patients over 60 years of age demonstrated a significant enhancement in mean eGFR with EMPA [85]. More recently, the CONFIDENCE trial revealed that simultaneous initiation of EMPA and finerenone resulted in a 29% and 32% greater reduction in UACR compared to finerenone or EMPA alone after 6 months, supporting the potential role of early combination therapy rather than sequential initiation in patients with albuminuric CKD and T2DM [86]. In Egyptian patients with CKD, EMPA demonstrated a marked reduction in proteinuria compared to placebo, with a median percentage change of − 80.9% versus − 48% (*P* < 0.001). Although improvements in eGFR and serum creatinine were not statistically significant, EMPA was associated with slower progression of renal impairment (hazard ratio 0.65; 95% CI 0.55–0.72; *P* = 0.002). These findings support the renal protective potential of EMPA in Egyptian patients with type 2 diabetes and CKD [87].

In 2024, KDIGO recommended initiation of EMPA in patients with eGFR ≥ 20 mL/min/1.73 m², and continuation even if eGFR falls below 20 mL/min/1.73 m² unless the drug is not tolerated or kidney replacement therapy is initiated [20]. The current evidence supports the renoprotective effect of EMPA in appropriate patients with CKD, but the extent of benefit may vary across patient populations and should not be interpreted as complete prevention of CKD progression.

### EMPA and weight loss

Obesity is a major health burden in Egypt, where approximately 32% of adults (44.7% of women, 25.9% of men) live with obesity (BMI ≥ 30 kg/m²) [88]. According to “100 million health” survey, which was conducted in Egypt in 2019 and screened 49.7 million adult Egyptians (≥ 18 years old), 39.8% of adult Egyptians suffered from obesity (BMI ≥ 30 kg/m^2^), with obesity was more prevalent in adult females than adult males (49.5% of Egyptian adult females suffered obesity compared to 29.5% for males) [89]. In a national survey of ever-married Egyptian women of reproductive age, obesity prevalence was 41.1% versus 22.7% in men [90].

EMPA’s weight-reducing effects may therefore have particular relevance in Egyptian and regional populations. In an Egyptian randomized trial in T2DM patients inadequately controlled on metformin, EMPA significantly reduced body weight compared with sitagliptin over 12 weeks [19].

Weight reduction demonstrated efficacy in improving diabetic symptoms during early disease stages [91]. The largest randomized controlled trial evaluated the weight management effect on cardiovascular disease risk in overweight T2DM patients, revealing decreased comorbidities [92]. In Egyptian patients treated with EMPA, there was a significant reduction in body weight, decreasing to 91.9 ± 18.0 kg after 12 weeks of therapy (*P* = 0.001), reflecting its beneficial effect on weight management alongside glycemic control [19].

A growing body of evidence has emphasized the key role of EMPA in decreasing body weight [93–98]. In fact, EMPA induces body weight loss at any dose regimen [93]. It also showed a significant decline in body fats, visceral fats, and leptin levels [99]. Neeland et al. revealed significant weight loss, waist circumference drop, and a decrease in adiposity indices regardless of age, sex, and waist circumference [100]. EMPA also showed a negative impact on muscle mass in another study [62]. In overweight and obese women with polycystic ovary syndrome, Javed et al. found reduced hip circumference and basal metabolic rate after 12 weeks of EMPA [95]. This may be related to transient body water loss following increased plasma renin activity with SGLT-2i [96].

### EMPA in patients with T1DM

Although evidence supports SGLT-2i efficacy in T1DM, they are not approved for this indication due to diabetic ketoacidosis (DKA) risk. Three large clinical trials reported serious DKA cases with SGLT-2i use in T1DM [101–103], with a recent meta-analysis estimating a twofold higher risk of DKA upon its use as an adjunct to insulin [104]. Consequently, both the European Medicines Agency (EMA) and the US Food and Drug Administration (FDA) retracted the temporary recommendation to use SGLT-2i as an adjuvant therapy to insulin in adult patients with T1DM [105]. Nevertheless, the risk of DKA was higher when hyperglycemia did not exist [106]. DKA in T1DM may follow severe illness, reduced food intake, mild infection, increased physical activity, or insulin dose reduction, though some cases lack identifiable triggers [107].

In combination with insulin, SGLT-2i in T1DM lowers blood glucose with minimal hypoglycemia risk, reduces glomerular hyperfiltration, lowers BP and volume overload, and promotes weight loss [107]. EMPA demonstrated positive effects on mean HbA1C, fasting glucose, and symptomatic hypoglycemia among patients with T1DM, resulting in a significant decrease in daily insulin dose [108–110]. The EASE Trials [111] and EASE-1 [112] studies revealed a significant decrease in mean body weight and fasting blood glucose levels during use of EMPA as adjunctive to insulin therapy.

SGLT-2i may benefit T1DM patients with obesity, insulin resistance, metabolic syndrome, hypertension, albuminuria, or poor time-in-range control. Use should be avoided in elderly patients, those non-compliant with ketone monitoring, those with uncontrolled diabetes, recurrent DKA, very low Body Mass Index (BMI), low insulin doses, or frequent severe infections [113]. This contradictory evidence signifies the need for large-scale controlled trials to assess the efficacy and safety of these drugs in patients with T1DM.

### EMPA in MASLD

A recent meta-analysis estimated the prevalence of MASLD, previously known as NAFLD, in the MENA region at 39.4% and almost 70% in patients with T2DM. The study suggests that Egypt exhibits one of the highest MASLD/NAFLD burdens in the MENA region, with an estimated 25.71 million affected individuals and a national prevalence of 45.0% [13]. Recent local data among Egyptian university students revealed a MASLD prevalence of 12.9% (by ultrasound plus metabolic criteria), with 57.8% of those affected being overweight or obese [114].

The American Association of Clinical Endocrinology (AACE) recommends SGLT-2i as adjuvant therapy in diabetic patients with MASLD [115]. A large body of evidence has proven efficacy in decreasing hepatic steatosis with improved cardiometabolic and kidney functions [12, 116]. SGLT-2i improves insulin sensitivity and reduces hepatic white adipose tissue inflammation by enhancing lipolysis and β-oxidation of free fatty acids, and lowering oxidative stress, inflammation, and apoptosis [117]. EMPA reduces ALT and AST levels while improving fasting glucose and HbA1c [118–121]. A potential positive impact of EMPA in improving systolic BP and reducing Body Mass Index (BMI) was also recorded in patients with liver diseases in the literature [122, 123], in addition to significantly decreasing steatosis and fibrosis in patients with DM [16, 122, 123]. Nevertheless, another study revealed a positive effect of EMPA on liver fat with or without DM [124]. A significant improvement in liver stiffness measure (LSM) and controlled attenuation parameter in patients on EMPA compared with pioglitazone was revealed [125]. A recent network meta-analysis comparing the effects of 26 hypoglycemic agents for treating MASLD identified EMPA as the most effective in reducing LSM [126], highlighting the promising role of EMPA in improving liver dysfunction in patients with DM. However, further clinical trials are warranted to confirm the efficacy and safety of EMPA in patients with MASLD with or without diabetes.

Evidence from Egypt provides notable support for EMPA’s effect on MASLD. In a study of 30 patients with T2DM and hepatic steatosis, EMPA 10 mg daily was added to standard care. After 24 weeks, mean hepatic fat fraction declined from 15.29 ± 8.72 to 13.30 ± 7.15, representing a 13.2% relative reduction (*P* = 0.006). Significant decreases in BMI, fasting glucose, and ALT were also observed, whereas Fib-4 and NAFLD fibrosis scores remained unchanged [17]. In nondiabetic Egyptian patients with metabolic fatty liver disease, a parallel open-label study (*n* = 55) showed that EMPA 10 mg plus lifestyle advice led to a mean CAP (controlled attenuation parameter) reduction of 38 dB/m vs. 8 dB/m in the lifestyle-only arm over 6 months (*P* = 0.001). Additional improvements were seen in weight, waist circumference, and BP [127].

### EMPA in Ramadan

During the holy month of Ramadan, Muslims at the age of puberty and onward are forbidden to drink or eat from dawn to sunset [128]. Sudden changes in nutrition, sleeping patterns, physical activity, and mealtimes can occur throughout Ramadan. These changes may increase the risk of metabolic complications in diabetics, including DKA, hypo- and hyperglycemia, and dehydration. The International Diabetes Federation (IDF) and Diabetes and Ramadan (DaR) Alliance recommend patient education on fasting and potential side effects during Ramadan [129]. Similarly, T2DM patients on EMPA who intend to fast during Ramadan should receive education on recognizing symptoms of hypoglycemia and dehydration at least six months before the commencement of fasting [129].

One study found more hypoglycemia episodes during Ramadan in SGLT-2i users on high insulin doses compared with other oral agents. Dehydration signs occurred in 9.3% of participants. However, weight and HbA1c dropped dramatically by the end of Ramadan, with no considerable changes in lipid profile or creatinine levels [130]. Other studies reported no increased risks of hypoglycemia, ketosis, or dehydration, along with weight loss and stable eGFR, among Pakistani, Bangladeshi, and Malaysian Muslims [131–134]. In another study, hypoglycemic symptoms during Ramadan were lower in patients who received EMPA compared to the control group among Egyptian Muslims [135]. During Ramadan, 300 Egyptian adults with type 2 diabetes participated in a multicenter study comparing EMPA (SGLT2 inhibitor) to sitagliptin (DPP-4 inhibitor), both taken with metformin. In the EMPA group, the average HbA1c dropped significantly (*p* = 0.021), while the sitagliptin group showed a smaller, non-significant change (*p* = 0.365). Both groups lost weight, but the reduction was greater with EMPA, a highly significant difference (*P* < 0.001). Only 6 patients (3.8%) on EMPA and 11 patients (7.7%) on sitagliptin reported mild side effects such as urinary or genital infections, and no cases of diabetic ketoacidosis occurred. These findings suggest that EMPA is a safe and effective option for Egyptian patients with T2DM fasting during Ramadan, helping improve glucose control and support weight loss with minimal adverse effects [136]. This variability may reflect differences in cultural and nutritional habits across Islamic countries.

Current evidence suggests that EMPA may be used safely by some patients with type 2 diabetes who fast during Ramadan, particularly when treatment is individualized, and patients receive appropriate education on hydration, recognition of adverse symptoms, and situations in which fasting should be interrupted. However, the available evidence remains limited by relatively small sample sizes, heterogeneous study designs, and restricted representation of high-risk patients. Treatment decisions should be individualized according to baseline glycemic control, kidney function, concomitant medications, volume status, and the individual’s overall risk of dehydration or ketoacidosis. Larger prospective studies, particularly in Egyptian and other Middle Eastern populations, are needed.

### Safety of EMPA

The safety of EMPA is well-established in various studies. Pooled analyses support the favorable safety and tolerability profile of both 10 and 25 mg doses [39]. A meta-analysis conducted on the four major clinical trials involving EMPA, including 19,727 patients, revealed the safety of EMPA in elderly patients with HF, reduced eGFR, and low BMI [137]. A single EMPA dose of 25 mg along with a 200 mg supratherapeutic dose could not change the heart rate-corrected QT interval following four hours of therapy [138]. Additionally, one study found no significant interaction with the simultaneous administration of other glucose-lowering medications or cardiovascular agents with SGLT-2i. Mutually, no changes in the pharmacokinetic measures of EMPA were detected, indicating predictable efficacy and safety of the drug [139]. The most commonly reported adverse events (AEs) are genital mycotic infections and urinary tract infections. Euglycemic diabetic ketoacidosis and Fournier’s gangrene remain rare but serious AEs [94]. International studies reported hypoglycemia occurring primarily in regimens combined with insulin or sulfonylureas rather than from EMPA itself. Overall, the robust clinical evidence highlights EMPA as a well-tolerated therapeutic option that provides meaningful metabolic and cardiovascular benefits for patients with type 2 diabetes [37, 38].

In a 12-week Egyptian study comparing EMPA and sitagliptin as add-on therapies to metformin, EMPA demonstrated a favorable safety profile with most adverse events being mild to moderate in severity [19]. Overall adverse event rates were comparable between groups (11.8% vs. 8.2%), with no reported serious complications of diabetic ketoacidosis or death. The most commonly reported events included genitourinary infections and mild dehydration. Five patients (5.9%) discontinued EMPA due to urinary tract infections, likely related to its glucosuric mechanism. Mild hypoglycemia occurred in only one patient (1.2%) on EMPA, with no severe episodes. Other events, such as headache or nasopharyngitis, were infrequent and did not require discontinuation. EMPA demonstrated a favorable safety profile consistent with international findings, supporting its safe use in type 2 diabetic patients inadequately controlled on metformin in Egypt [19].

### Cost-effectiveness with EMPA

In 2018, EMPA increased lifetime treatment costs compared with standard care, while improving Quality-Adjusted Life Years (QALYs) [140]. At an added cost of €4235, EMPA extended mean survival to 14.01 years versus 11.87 years with standard care and yielded 7.75 QALYs versus 6.83 [141]. Based on EMPA-REG and CANVAS, the estimated Per Member Per Year (PMPY) cost for Major Adverse Cardiovascular Events (MACE), including CV death, nonfatal MI, nonfatal stroke, and HF hospitalization, was $6.17 (range $1.27–$10.94) for CANVAS and $2.75 ($0.19–$4.83) for EMPA-REG [142]. Another study revealed an increment in survival of 2.1 life-years in diabetic patients with CVDs while using EMPA compared to standard of care [143].

Furthermore, based on EMPA-REG outcomes and cardiovascular outcomes trials, specific treatment effects on CVD risk factors, EMPA, in addition to standard of care, were cost-effective in comparison with sitagliptin or saxagliptin, each administered with standard of care [27]. Moreover, using EMPA for the prevention of CV death or HF hospitalization in T2DM patients demonstrated superior cost-effectiveness compared to canagliflozin and dapagliflozin [28]. In the UK, EMPA (25 mg) plus metformin costs approximately £6000 less than semaglutide (14 mg) plus metformin for T2DM treatment [29]. A large US claims-based comparative effectiveness study found EMPA to be associated with lower total healthcare costs (ranging from -$847 to -$1425) driven by pharmacy costs when compared to GLP-1 agonists. However, no difference was detected in hospitalization or Emergency department utilization [144].

Based on the EMPA-KIDNEY trial, the use of EMPA was associated with a significant reduction in CKD progression and overall health costs [145]. In patients with CKD, in comparison to standard of care alone, EMPA with standard of care was linked to greater health benefits of 11.06 life years and 9.01 QALYs [146]. Nevertheless, studies found that semaglutide showed superior cost-effectiveness in Spain [147] and Thailand [148] compared to EMPA for T2DM patients with uncontrolled blood glucose levels, suggesting that the total costs of these treatments should be considered for cost-effectiveness. However, evidence of the cost-effectiveness of EMPA in the local and regional context is still lacking.

Although international studies suggest that empagliflozin may be cost-effective in selected populations, their findings cannot be directly extrapolated to Egypt because cost-effectiveness depends on local drug prices, healthcare costs, financing, reimbursement, clinical risk, and willingness-to-pay thresholds. In Egypt, its potential economic value is substantial given the burden of diabetes, cardiovascular disease, and chronic kidney disease; however, limited local pharmacoeconomic data make the magnitude of this benefit uncertain. Therefore, international evidence should be interpreted cautiously, and future Egyptian studies should incorporate local costs, healthcare utilization, renal replacement therapy, clinical outcomes, and willingness-to-pay thresholds.

This review has several limitations. First, as a comprehensive narrative review, it does not employ a formal systematic-review protocol or standardized quantitative risk-of-bias assessment. Second, the quantity and quality of evidence vary substantially across the clinical applications discussed. Evidence supporting cardiovascular and renal benefits is more robust than evidence related to MASLD, type 1 diabetes, Ramadan fasting, and local cost-effectiveness. Third, relatively few studies have been conducted specifically in Egyptian populations, limiting the ability to draw country-specific conclusions.

## Conclusion

This comprehensive review sheds light on the crucial effects of EMPA in regulating renal and cardiovascular disease progression in patients with or without diabetes, with particular attention to the Egyptian population, where the burden of T2DM, ASCVD, MASLD, and CKD is high and projected to grow. Evidence from Egyptian cohorts and Clinical trials supports EMPA’s efficacy and safety in this setting, reinforcing its role in improving glycemic control, cardiovascular and renal outcomes, and QoL. However, future large-scale studies are needed in Egypt to assess its safety, efficacy, and cost-effectiveness in special populations, including MASLD, Muslims during fasting, and patients with T1DM, and to address the current gap in the local health-economic data.

## Data Availability

No datasets were generated or analysed during the current study.
